# An Exploration into the Origins and Pathogenesis of Anaplastic Large Cell Lymphoma, Anaplastic Lymphoma Kinase (ALK)-Positive

**DOI:** 10.3390/cancers9100141

**Published:** 2017-10-24

**Authors:** Suzanne D. Turner

**Affiliations:** Division of Cellular and Molecular Pathology, Department of Pathology, University of Cambridge, Cambridge CB2 0QQ, UK; sdt36@cam.ac.uk; Tel.: +44-122-376-2655

**Keywords:** ALCL, ALK, thymus, lymphoma

## Abstract

T-cell non-Hodgkin lymphoma is a heterogeneous disease ranging from malignancies arising from thymic T cells halted in development, through to mature, circulating peripheral T cells. The latter cases are diagnostically problematic with many entering the category of peripheral T-cell lymphoma, not otherwise specified (PTCL, NOS). Anaplastic large cell lymphoma (ALCL) is one of the exceptions to this whereby aberrant expression of anaplastic lymphoma kinase (ALK) and the distinctive presence of cell surface CD30 places this entity in its own class. Besides the expression of a well-studied oncogenic translocation, ALCL, ALK+ may also have a unique pathogenesis with a thymic origin like T lymphoblastic lymphoma but a peripheral presentation akin to PTCL. This perspective discusses evidence towards the potential origin of ALCL, ALK+, and mechanisms that may give rise to its unique phenotype.

## 1. Introduction

Systemic anaplastic large cell lymphoma (ALCL) is a relatively rare malignancy of T cells and is sub-divided dependent on expression of anaplastic lymphoma kinase (ALK) creating ALCL, ALK+ and ALCL, ALK− sub-classes [1,2]. The former is largely diagnosed in younger patients, has a good prognosis and is addicted to ALK, whilst the latter is seen in an older patient demographic with a relatively poor prognosis and a scarcity of driving oncogenic events. ALCL can also present as a cutaneous form or in the context of breast implants, both being ALK negative [3,4]. Given the various presenting forms and differing prognoses, it makes sense that the ALCL sub-types should form distinct disease categories but also raises the issue of whether they are the consequence of a shared pathogenesis and origin. Indeed, mounting evidence points towards a thymic or more primitive haemopoietic origin for ALCL, ALK+ with perhaps in utero derivation akin to some childhood leukaemia, in contrast to a putative peripheral and adulthood initiation of ALCL, ALK− [5,6,7]. However, it is likely that there are overlapping mechanisms of pathogenesis given their shared and unique histopathology. This perspective will discuss and present evidence eluding towards the aforementioned mechanisms, comparing and contrasting disease processes with reference to the relative merits of model systems and techniques that have been employed to divulge this information.

## 2. T-Cell Development and the Origins of ALCL

Solid cancers of T cells are considered as having either mature or immature origins dependent on a number of diagnostic factors, ultimately presenting as either immature or mature T-cell lymphoma located in the thymus or at nodal/extranodal sites in the periphery. A thymic origin is reserved for T lymphoblastic lymphoma (T-LBL) whereby tumours can present in the thymus and/or periphery and may even have a leukaemic presentation, but T-cell receptor (TCR) rearrangements are in all cases supportive of a primitive origin [8,9,10]. In contrast, ALCL is considered a peripheral T-cell lymphoma due to the expression of mature, activated T cell markers as well as largely peripheral presentation of tumours. However, in the absence of ALK expression, diagnosis can be difficult and may overlap with peripheral T-cell lymphoma, not otherwise specified (PTCL, NOS) although expression of CD30 and key histological features enable better distinction as can the gene expression profile [11,12,13]. Regardless, broadly speaking, if a T-cell malignancy shares features with an immature T cell, it is called a lymphoblastic lymphoma, thought to arise from thymic T cells with all others presumed to derive from peripheral T cells. Whilst this is an easy way to define malignancies, one must consider that the final form in which a malignancy presents may mask its natural history.

### 2.1. T-Cell Receptor Gene Rearrangement Status Presents a History and Time Stamp of Thymocyte Development

Like B cells, T cells uniquely rearrange antigen receptors which provide a tattoo of their progression through the thymus; thymic progenitors rearrange the TCR genes in a temporal and location-specific manner on encountering neighbouring, thymic-resident cells presenting antigen in an MHC-restricted manner [10,14]. Ultimately, successful rearrangement leads to the emergence of T cells from the thymus that recognise foreign antigens presented together with MHC, but not self-proteins. Most commonly, T cells leave the thymus with rearranged α and β chains to become αβ T cells, whilst some emerge as γδ T cells having not progressed to rearrangement of α and β chain genes. As such, T cells only emerge into the periphery if these processes are successful except in cases where thymocyte survival is subverted by the presence of TCR-bypass events. As such, analysis of TCR rearrangement status in malignancies of these cells can provide clues as to their origin and the stage of T cell development they reached prior to transformation [10].

### 2.2. TCR Rearrangements in ALCL are Suggestive of Stalled Thymocyte Development of Apparently Mature T Cells Indicative of a Primitive T-Cell Origin for this Malignancy

ALCL is considered a T-cell lymphoma even though tumour cells often lack expression of cell surface proteins indicative of this cellular phenotype. However, molecular TCR rearrangements are detectable in the tumour cells supportive of a T-cell origin. Indeed, an in-depth analysis of these molecular rearrangements in ALCL, ALK+ has highlighted some unusual *T cell receptor* (*TR*) genotypes not normally permissive of thymocyte survival and T cell development [6]: an analysis of 57 ALCL showed that whilst 56% of tumours display apparently normal *TRab* rearrangements, 82% of these 56% do not have major clonal *TRb* rearrangements. In addition, 11% of tumours only have *TRg* rearrangements, and 14% have no *TR* rearrangements at all consistent with a null cell immunophenotype. The remaining 19% could potentially originate in normal γδ T cells with apparently normal *TRg* and *TRd* rearrangements. Overall, these data are supportive of a consistent lack of major clonal *TRb* rearrangements in ALCL, suggesting that thymic processes may have been perturbed in these patients; expression of a strong survival factor may allow T cells without survival-permissive TCR to develop and escape into the periphery. Indeed, in a murine model, NPM-ALK has the capacity to enable this, whereby, even in the absence of RAG, the enzyme responsible for this process, T-cell development appears normal [6]. This is perhaps not surprising given the plethora of mitogenic/survival signaling pathways known to be activated by this oncogene including those normally active as a consequence of TCR engagement [15,16].

Intriguingly and together with the detection of NPM-ALK in cord blood of 2% of the healthy population [17], these data raise the possibility that like T-LBL, ALCL is also a malignancy arising in thymocytes but in the latter case, thymocytes that can still apparently progress through T-cell developmental stages in the absence of *TR* rearrangement [6]. Alternatively, the translocation may be induced at a more primitive stage, as suggested by the findings in cord blood although this then raises the question as to why NPM-ALK is restricted to T-cell lymphoma as opposed to any other malignancy derived from haemopoietic stem cells.

## 3. NPM-ALK Induced Signaling Events May Counteract Thymic Beta-Selection

NPM-ALK is a hyperactive tyrosine kinase by virtue of its ability to dimerise and subsequently autophosphorylate on tyrosine residues [18]. These then form docking sites for SH2 domain containing proteins activating a plethora of signaling pathways largely including those expected of a hyperactive intracellular kinase [19]. For example, signaling through PI 3-Kinase-AKT, mTOR, RAS MAP Kinase, JNK-Jun, AP-1, JAK/STAT, and PLCg/Ca^2+^ pathways and transcription factors is well-established, all pathways with the potential to drive proliferation and promote cell survival, two key hallmarks of malignancies [15,20,21,22,23,24,25,26,27,28,29,30,31]. The first key checkpoint in thymic development is β-selection, a process whereby the absence of a signal emanating from an engaged pre-TCR leads to cell death. Cell survival is dependent on activation of Notch 1, and NPM-ALK is able to activate signaling via this pathway in murine thymocytes at the DN2/3 stages of thymic development [6]. Indeed, NPM-ALK expression in thymocytes also leads to the upregulation of CD98 and CD71, nutrient transporters required for massive cellular proliferation associated with post-β-selection thymocytes [6]. It follows that Notch 1 is expressed on the surface of established ALCL tumour cells, possibly a remnant of their time in the thymus [32]. Notch 1 is also known to play a role in mature, peripheral T cells [33], but the relative importance of Notch 1 to thymic development is underscored by the detection of Notch 1 mutations in thymic-derived T-ALL [34].

## 4. Accounting for the Activated Cellular Phenotype of ALCL

ALCL has an unusual presentation with an immunophenotype that does not place it neatly into any specific T cell subset. In general, most tumours are negative for CD8 but produce cytotoxic proteins such as perforin and Granzyme B, and whilst being largely CD4 positive, they lack expression of a TCR/CD3 [4,35]. Regardless, expression of CD30 is consistent but does not confer a lineage identity to the cells beyond their apparent activation status [4,36]. Indeed, tumour cells also express the epithelial cell protein EMA (MUC1), expression of which has also been linked to activated T cells [37,38]. However, as mentioned above, if NPM-ALK can substitute for pre-TCR signaling in thymocytes, then it can also potentially mimic signals indicative of an activated status normally observed on engagement of a full TCR [15]. Beyond these lineage-defining cell surface proteins, molecular TCR rearrangements are supportive of a T-cell status [6].

### 4.1. Is NPM-ALK, the Tumour Microenvironment, and/or the Cell of Origin Responsible for the Immunophenotype of Cells in ALCL, ALK*+*?

**Figure 1:**
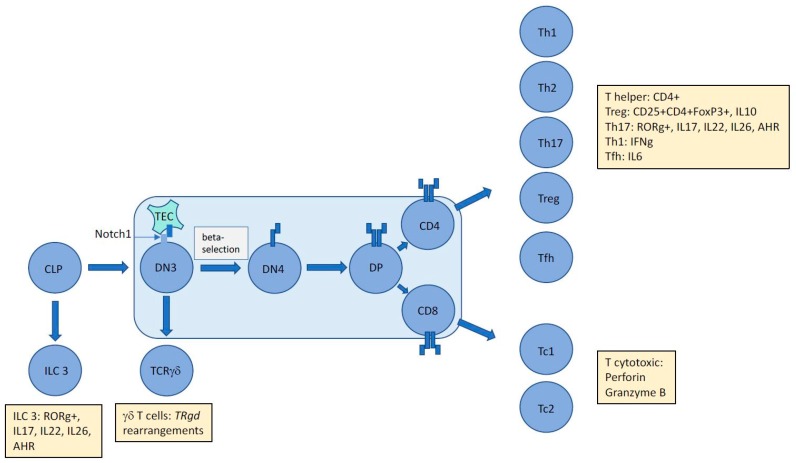
The unique immunophenotype of ALCL makes it difficult to assign an exact cellular origin with gene expression studies unable to divulge a distinct cell of origin. The presence of aberrant TCR rearrangements suggests subversion of thymic development mediated by NPM-ALK in a Notch1-dependent manner proposing an early origin for this largely paediatric cancer. However, tumour cells express and secrete proteins (denoted by yellow boxes) that inhibit assignment to an exact T cell subset. Whether these proteins are induced by driving oncogenic events and/or the tumour microenvironment remains to be determined, but in the case of ALCL, ALK+ at least, it is clear that NPM-ALK is capable of partially mimicking the immunophenotype of multiple T-cell subsets. CLP = common lymphoid progenitor; DN = double negative; DP = double positive; TEC = Thymic Epithelial Cell; ILC = Innate Lymphoid Cell; AHR = Aryl Hydrocarbon Receptor; Th = helper T cell; Tc = cytotoxic T cell; Treg = regulatory T cell; Tfh = follicular helper T cell; *TR* = T cell receptor.

It is possible to garner more information on a cell’s identity by its secretome, particularly in the T cell lineage, whereby distinct cytokines can both induce a skew towards a specific effector cell type and likewise, when secreted can divulge the identity of the producing cell, as can the distinct profile of transcription factors active within it. Hence, even though primitive forms of gene expression profiling have been unable to align ALCL with either cytotoxic or helper T cells, some enriched gene sets have highlighted specific associations [36]. For example, a Th17 profile could be extracted whereby expression of a set of genes including *IL17A*, *IL17F*, *IL26*, *IL22*, and RORg was noted [12]. Interestingly, IL22 and IL17 are also present in the circulation of ALCL, ALK+ patients [39]. Naturally, immune cells are heavily influenced by their microenvironment and cytokines within it that may have stimulatory effects on them. A Th17 skew is induced by IL6 in the presence of TGFb, and it follows that IL6 is produced in an autocrine manner by ALCL in an NPM-ALK dependent-manner via STAT3 and miR135b [40]. This oncogene-driven pathway was also shown to be responsible for the production of IL17A and IL17F, suggesting that the Th17 phenotype could be attributable to NPM-ALK expression and activity rather than microenvironmental factors or even cell of origin [40]. Furthermore, perforin and Granzyme B are produced in an NPM-ALK-dependent manner again providing evidence towards a cell phenotype modelled by the driving oncogene [40,41]. Of note, in the study by Matsuyama et al, the Th17-associated transcription factor RORg was not dependent on miR-135b activity although it remains to be determined whether it is expressed in an NPM-ALK-dependent manner [40]. Altogether, these data suggest that the immunophenotype of ALCL, ALK+ is not incompatible with a thymic origin, whereby NPM-ALK is able to drive thymic development in the absence of complete and survival-compatible TCR rearrangements to allow cells to ‘mature’, enter the periphery, and appear to be of a Th17/cytotoxic T-cell origin (Figure 1).

### 4.2. What Shapes the Phenotype of ALCL, ALK−?

Whether a similar scenario applies to ALCL, ALK− remains to be determined, but as oncogenic drivers are identified for this heterogeneous category, a similar picture emerges. For example, miR155 is upregulated in ALCL, ALK−, a miR that is known to induce a Th17 cell skew, and despite some cases displaying JAK1/STAT3 mutations, cells remain cytokine-dependent [42,43,44]. Whilst it can be difficult to tease apart mechanisms specific to ALCL, ALK− due to a relative paucity of model systems, the more recent description of breast-implant-associated ALCL (BIA-ALCL) has provided more clues [3,45,46]. Cell lines derived from BIA-ALCL and cutaneous ALCL also produce IL17A, IL17F, and IL6, but IL10, IL13, IL21, IFNg, GM-CSF, IL7, and TNFa are also produced, cytokines that are attributable to other helper T cell subsets [44]. However, the presence of activating JAK/STAT mutations in BIA-ALCL may also be driving this particular secretome [47,48]. Indeed, ALCL, ALK− are also associated with activating mutations/translocations in JAK/STAT family proteins [49] and 4% of cutaneous ALCL with TYK2 fusion proteins [50]. Could it be the case that each individual case of ALCL has a unique profile that is shaped not only by the intrinsic oncogenic events but also its particular microenvironmental circumstance? Th17 cells are known to react to large extracellular pathogens such as bacteria, whereas Th1 cells, for example, respond to intracellular pathogens. Bacterial biofilms have been reported in the context of BIA-ALCL, which fits with this hypothesis and would also give rise to an activated cell surface phenotype as exemplified by CD30 expression [51]. In this regard, ALCL have been reported to be associated with tick and other insect bites, which could lead to infection [52,53].

### 4.3. Does Infection Play a Role in ALCL Lymphomagenesis and Cellular Immunophenotype?

The fact that the tumour cells appear to be activated by virtue of CD30 expression yet not having a gene expression profile compatible with an activated CD30+ T cell hints towards an oncogene-driven rather than environmental event [36]. Indeed, CD30 expression has been reported as an NPM-ALK driven JunB-induced event in ALCL [54]. Whilst this suggests that activation is not a consequence of antigenic stimulation, as mentioned previously, ALCL, ALK+ have been reported in the context of tick and other insect bites although in some cases, ALCL, ALK+ do not have the ability to express a functional TCR and proximal signaling proteins are silenced in an epigenetic manner [6,35,52,53,55]. However, some lymphoid cells exist that respond to inflammatory environments in a receptor-independent manner. These cells are the relatively recently described innate lymphoid cells (ILCs) of which there are three distinct groups with origins in common lymphoid progenitors. Of interest, Group 3 ILCs share many facets with Th17 cells: expression of RORg and AHR as well as production of IL17 and IL22 [56]. It may therefore be the case that incipient ALCL without functional TCR responds to infections akin to Group 3 ILCs, whereas those with a TCR act as Th17 or as another T-cell subset. In the latter case, the TCR is subsequently downregulated as results from a murine model show that a combination of signaling through a TCR and NPM-ALK are not compatible with lymphomagenesis, and the TCR is rarely expressed in primary ALCL [6,35].

## 5. Conclusions

ALCL and other T-cell lymphomas remain malignancies of unknown origins and causes with few aetiological associations. This is largely due to a lack of model systems and a relatively scarcity of patient material from which to divulge information. However, marrying together clinical observations with laboratory studies has allowed some progress to be made.
